# Conserved water molecules shape the pathogenicity of missense variants in human proteins

**DOI:** 10.1093/molbev/msag217

**Published:** 2026-11-04

**Authors:** Janez Konc, Karmen Recer, Tanja Kunej, Dušanka Janežič

**Affiliations:** Theory Department, National Institute of Chemistry, Ljubljana SI-1001, Slovenia; Faculty of Mathematics, Natural Sciences and Information Technologies, University of Primorska, Koper SI-6000, Slovenia; Faculty of Pharmacy, University of Ljubljana, Ljubljana SI-1000, Slovenia; Theory Department, National Institute of Chemistry, Ljubljana SI-1001, Slovenia; Biotechnical Faculty, Department of Animal Science, University of Ljubljana, Domžale SI-1230, Slovenia; Biotechnical Faculty, Department of Animal Science, University of Ljubljana, Domžale SI-1230, Slovenia; Faculty of Mathematics, Natural Sciences and Information Technologies, University of Primorska, Koper SI-6000, Slovenia

**Keywords:** missense variants, pathogenicity, conserved water molecules, human structural proteome, variant interpretation

## Abstract

Conserved water molecules (CWMs) are tightly bound solvent molecules that occupy well-defined, recurrent positions in protein structures. Although they are known to influence protein stability, function, and ligand binding, their role in shaping the effects of human missense variants remains largely unexplored. Here, we demonstrate that CWMs are a previously underappreciated determinant of missense variant pathogenicity. By predicting ligand-binding and CWM sites across human PDB structures and mapping missense variants to these sites and the remaining protein surface, we found that pathogenic variants were significantly enriched at CWM sites, whether overlapping or outside other ligand-binding regions. This enrichment exceeded that observed for binding sites as a whole, indicating a broader role for water-mediated interactions in modulating variant effects. To explore a mechanistic basis for this association, we performed molecular dynamics simulations of human lysosomal acid glucosylceramidase (GCase), encoded by *GBA1* and implicated in Gaucher disease and Parkinson's disease risk. Selective destabilization of a CWM site in wild-type GCase produced structural and dynamical changes resembling those observed in the pathogenic L444P variant, whereas stabilization of this site in L444P shifted several measures toward wild-type behavior. These results suggest that disruption of a single CWM can contribute to long-range structural remodeling observed in a disease-associated variant. Together, our findings identify CWMs as a novel structural constraint shaping the distribution and effects of pathogenic missense variants. Incorporating water-mediated interactions into structural models provides a generalizable framework for interpreting human genetic variation and its contribution to disease.

## Graphical Abstract

**Graphical Abstract:**
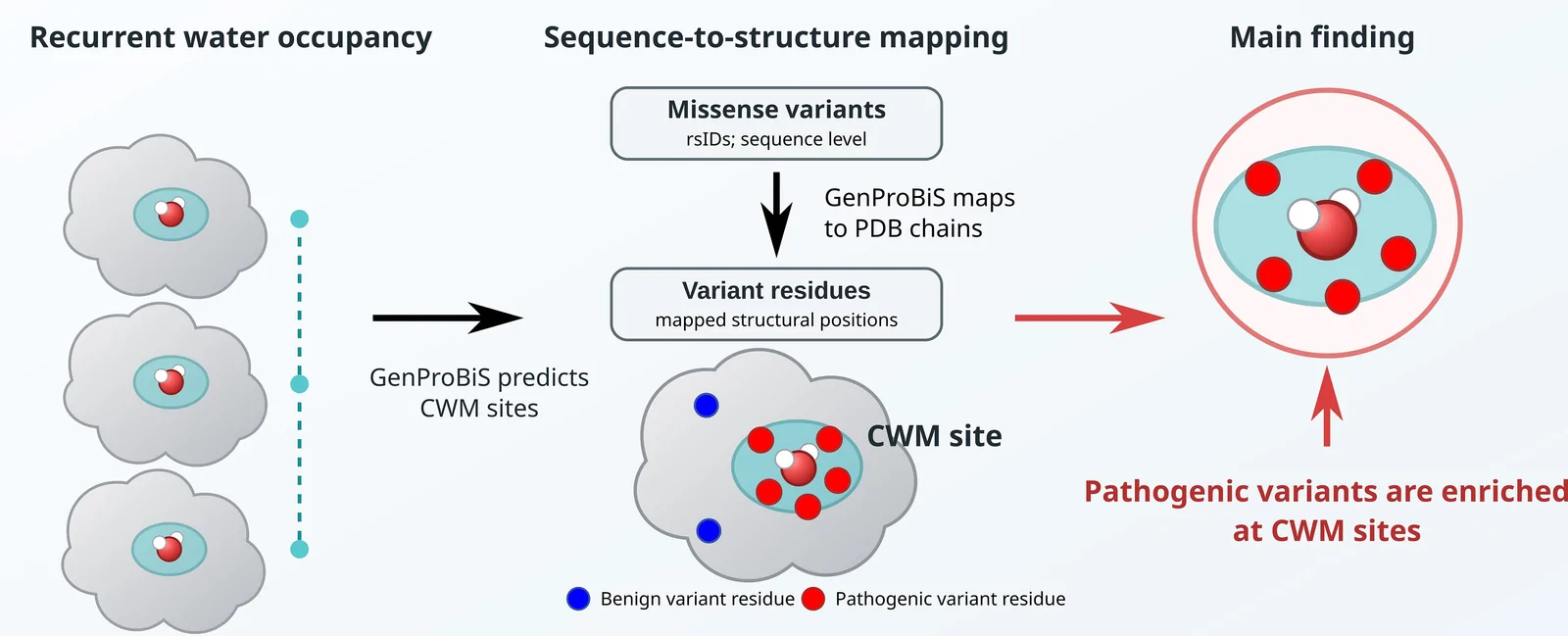

## Introduction

A central challenge in human genomics is to explain why mutations at similar structural locations within a protein can have markedly different functional consequences. Conserved water molecules (CWMs) are specific solvent molecules that form persistent, geometrically constrained interactions that stabilize protein structure and mediate ligand recognition (Shaltiel et al. 1998; Knight et al. 2009; Ball 2017; Spyrakis et al. 2017; Setny 2020; Campomizzi et al. 2024); yet it remains unclear whether they systematically influence the effects of genetic variation.

Proteins interact with diverse ligands, including proteins, nucleic acids, metal ions, cofactors, small molecules, and CWMs. Missense variants frequently perturb protein binding sites, disrupting ligand recognition or local stability (Lander et al. 2001; Auton et al. 2015; Kucukkal et al. 2015; Backwell and Marsh 2022). Structural information provides a powerful framework for interpreting these effects (Jubb et al. 2017; Sivley et al. 2018), supported by expanding coverage of experimentally determined structures in the Protein Data Bank (PDB) (Burley et al. 2025) and high-accuracy computational models (Jumper et al. 2021; Abramson et al. 2024). These resources underpin large-scale efforts to interpret human variation (Cheng et al. 2023), alongside rapidly growing variant datasets from population and disease sequencing (Gibbs et al. 2003; Auton et al. 2015; Ruderfer et al. 2016; Karczewski et al. 2020; Taliun et al. 2021; Dyer et al. 2025; Phan et al. 2025).

Pathogenic variants are enriched in binding sites across multiple ligand classes, including protein–protein, nucleic acid, and small-molecule interfaces (David et al. 2012; Das et al. 2014; Rolland et al. 2014; David and Sternberg 2015; Kamburov et al. 2015; Sahni et al. 2015; Porta-Pardo et al. 2017; Meyer et al. 2018; Livesey and Marsh 2022; Burke et al. 2023). However, the molecular features that distinguish pathogenic from benign variants within these regions remain incompletely understood. In particular, CWMs have not been systematically evaluated in this context, despite case evidence linking disruption of conserved water-mediated interactions to disease (Rodríguez-Almazán et al. 2008). Through their structural roles, CWMs may represent a broader determinant of human missense variant pathogenicity.

To test this hypothesis, we used the GenProBiS web server to predict ligand-binding and CWM sites across human PDB structures and to map benign and pathogenic missense variants to these sites (Konc and Janežič 2010, 2012, 2014; Konc et al. 2017). GenProBiS establishes CWM conservation from recurrent water occupancy across locally aligned protein structures rather than from a single structure. Accordingly, the variants were mapped to amino-acid residues in protein structures (variant residues), without computationally introducing substitutions or directly comparing wild-type and variant structures. Pathogenic variants were significantly enriched at CWM sites, supporting a systematic association between water-mediated structural interactions and variant pathogenicity.

We further investigated the mechanistic basis of this association using the pathogenic L444P substitution in lysosomal acid glucosylceramidase (GCase), which causes Gaucher disease and increases Parkinson's disease risk (Dvir et al. 2003). Molecular dynamics simulations illustrated how disruption of a single CWM can contribute to structural changes extending toward the active-site entrance. These findings are consistent with and extend previous observations for L444P (Romero et al. 2019), while underscoring the need to consider conserved water-mediated effects in therapeutic development (Williams et al. 2024).

Together, our results identify CWMs as previously unrecognized structural determinants of missense variant effects. By demonstrating a systematic association with pathogenic variants and illustrating a plausible mechanism, we provide a generalizable framework for incorporating water-mediated interactions into the interpretation of human genetic variation. To support mechanistic studies, pathogenicity prediction, and drug discovery, we provide a freely available dataset mapping variants to CWM and other ligand-binding sites (Supporting Information, Dataset S1).

## Results

### Missense variant pathogenicity inside vs. outside binding sites

We analyzed 4,580 nonredundant missense variants mapped to protein structures in the PDB, classifying them as within binding sites (inside) or on nonbinding protein surfaces (outside) (see definitions in Methods) (Fig. 1a). Pathogenic missense variants were more frequent inside than outside binding sites (*n* = 1,958 vs. 1,257), with a higher overall missense variant count (*n* = 2,466 vs. 2,114) and a greater pathogenic fraction (pf 79.4% vs. 59.5%) (Fig. 1b and c). Missense variants in binding sites of any type were almost three times more likely to be pathogenic than those on nonbinding surfaces (odds ratio [OR] = 2.63; 95% confidence interval [CI] 2.31 to 3.00; *P* = 4.93 × 10^−49^) (Fig. 1d).

**Figure 1:**
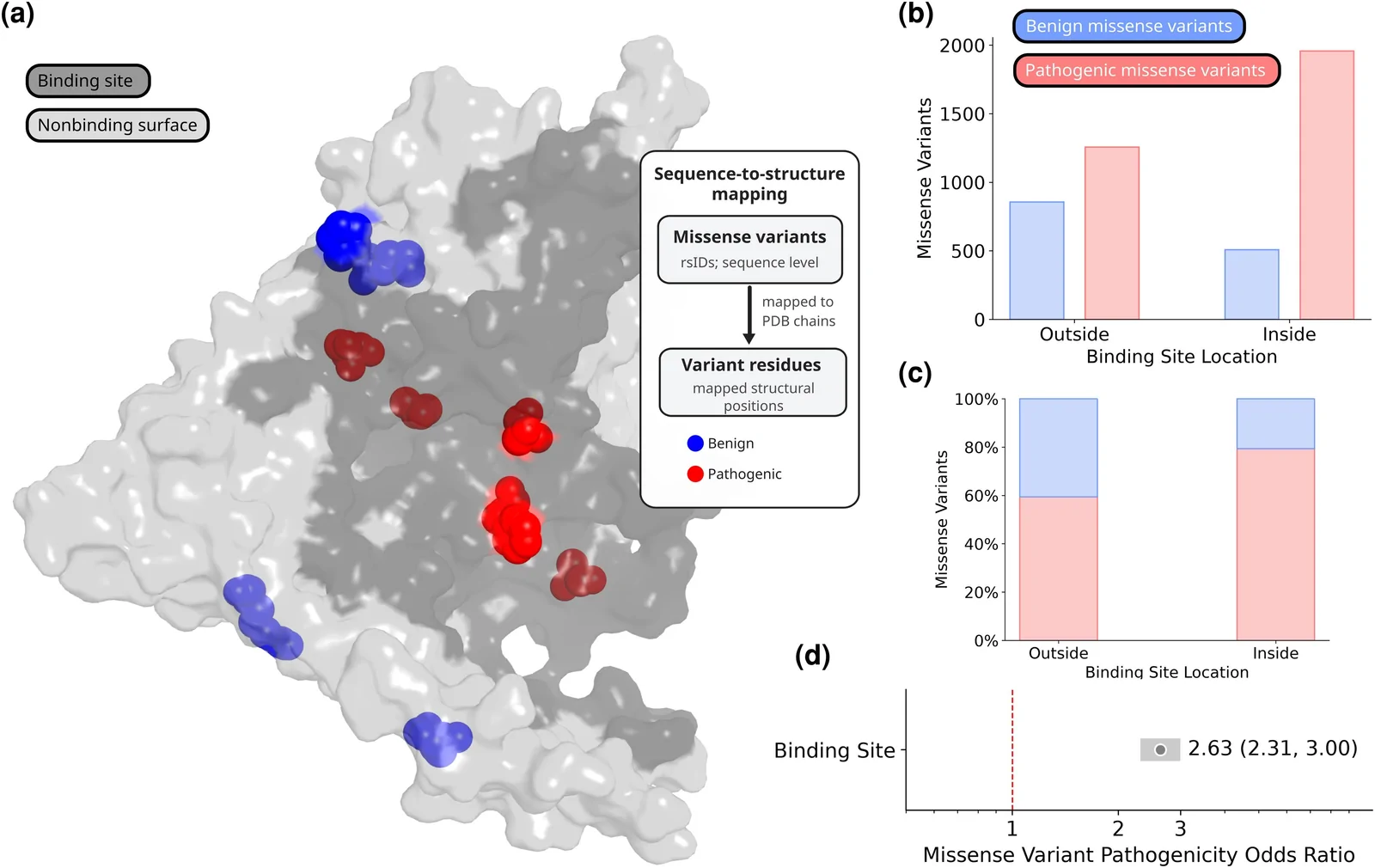
Pathogenic missense variants are enriched in protein binding sites. a) Pathogenic (red) and benign (blue) missense variants mapped to their corresponding protein residues (variant residues) in L-amino acid decarboxylase (*DDC*; PDB 3RBF). The dark-gray surface denotes the union of predicted binding sites for all considered ligand types, whereas the light-gray surface denotes the nonbinding protein surface. Variants were classified as inside when the mapped residue overlapped a predicted ligand-binding site and outside when it lay on the nonbinding surface (Methods). Reference SNP identifier, rsID. b) Counts of benign (blue) and pathogenic (red) missense variants outside versus inside binding sites. c) Percentage composition of benign and pathogenic missense variants in each location category (pathogenic fractions, red). d) Odds ratio (OR) and 95% confidence interval (CI) for pathogenicity among variants inside versus outside binding sites; grey point, OR; horizontal bar (light grey), CI; dashed vertical red line, OR = 1 (no association).

### Effect of binding site type on missense variant pathogenicity

Across binding site types (see examples in Fig. 2a), metal ion sites showed the strongest association with pathogenicity (OR = 8.52; CI 4.45 to 16.32; *P* = 2.66 × 10^−17^; pf = 92.6%; *n* = 125) (Fig. 2b and c). Other types were also significantly enriched (*P* < 10^−8^): cofactor (OR = 6.55; CI 4.64 to 9.25; *P* = 1.49 × 10^−38^; pf = 90.6%; *n* = 365), compound (OR = 4.10; CI 3.34 to 5.03; *P* = 2.6 × 10^−17^; pf = 85.7%; *n* = 782), nucleic acid (OR = 3.48; CI 2.14 to 5.66; *P* = 3.04 × 10^−8^; pf = 83.6%; *n* = 102), and protein (OR = 2.45; CI 2.12 to 2.83; *P* = 2.01 × 10^−36^; pf = 78.2%; *n* = 1,383), whereas glycan sites showed no significant enrichment (OR = 1.09; CI 0.81 to 1.47; *P* = 0.598; pf = 61.5%; *n* = 123).

**Figure 2:**
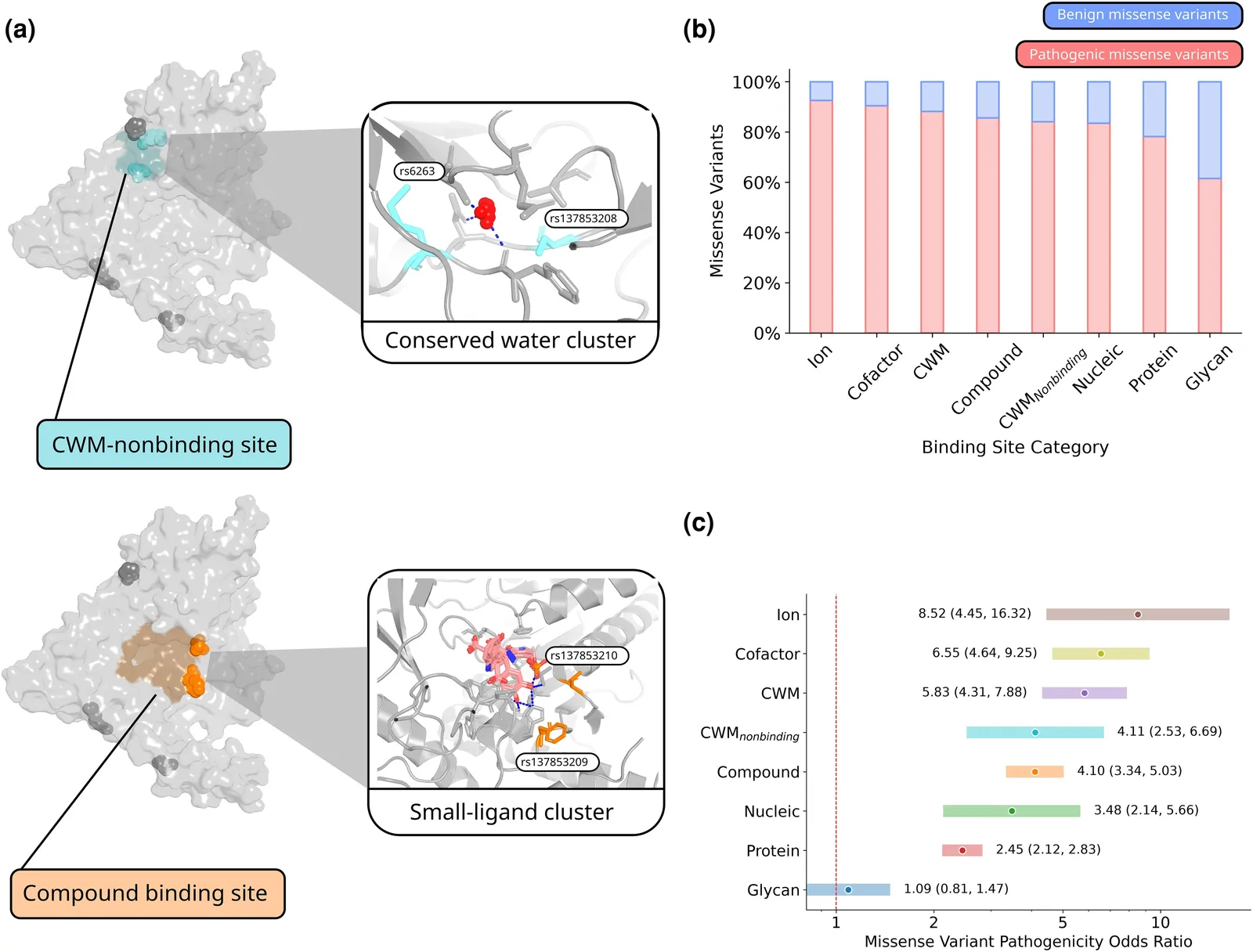
Binding site type-specific effects on missense variant pathogenicity. a) Missense variants mapped to variant residues (spheres) in CWM-nonbinding (cyan surface) and compound (orange surface) binding sites or to nonbinding surface (light-grey surface) in aromatic-L-amino-acid decarboxylase (*DDC*; PDB 3RBF). Insets show predicted ligands bound to the protein: water molecules (red spheres), compounds (pink sticks); hydrogen bonds between ligands and protein residues are shown as blue dashed lines; pathogenic variant residues (cyan and orange sticks) are labeled with their reference SNP identifiers (rsIDs). b) Percentage compositions of benign and pathogenic missense variants by binding site category (pathogenic fractions, red). c) Odds ratios and 95% CIs for the association between pathogenic missense variants and each binding site type; CWM and CWM-nonbinding site ORs calculated at water conservation level = 0.6; dashed red line, no association.

### Conserved water molecule (CWM) binding sites

To distinguish CWM sites by structural context, we analyzed two categories. “CWM sites” included all conserved water molecule sites, regardless of whether they overlapped another ligand-binding site. “CWM-nonbinding sites” were defined as the subset of CWM sites located on nonbinding protein surface, i.e. outside other predicted ligand-binding sites (see definitions in Methods). CWM sites were strongly associated with pathogenic missense variants (OR = 5.83; CI 4.31 to 7.88; *P* = 4.56 × 10^−41^; pf = 88.3%; *n* = 399); CWM-nonbinding sites retained substantial enrichment (OR = 4.11; CI 2.53 to 6.69; *P* = 1.53 × 10^−10^; pf = 84.1%; *n* = 106) (Fig. 2b and c).

### Water conservation and evolutionary conservation

In CWM sites, missense variant pathogenicity increased with water conservation, reaching OR = 14.72 at threshold 1.0 (*P* = 1.05 × 10−7), although estimates at high thresholds are limited by sample size (Fig. 3a). Evolutionary conservation of amino acid residues also correlated with pathogenicity both inside and outside binding sites (Fig. 3b and c): for residues at the highest conservation level of 9, 94.4% of binding-site variant residues and 81.5% of nonbinding surface variants were pathogenic, compared with 73.1% and 51.2% at level zero. Protein–protein sites showed the clearest monotonic increase in pathogenicity with conservation; similar trends were observed in CWM and compound sites, though patterns in other types were less consistent due to small numbers (SI, Figure S1).

**Figure 3:**
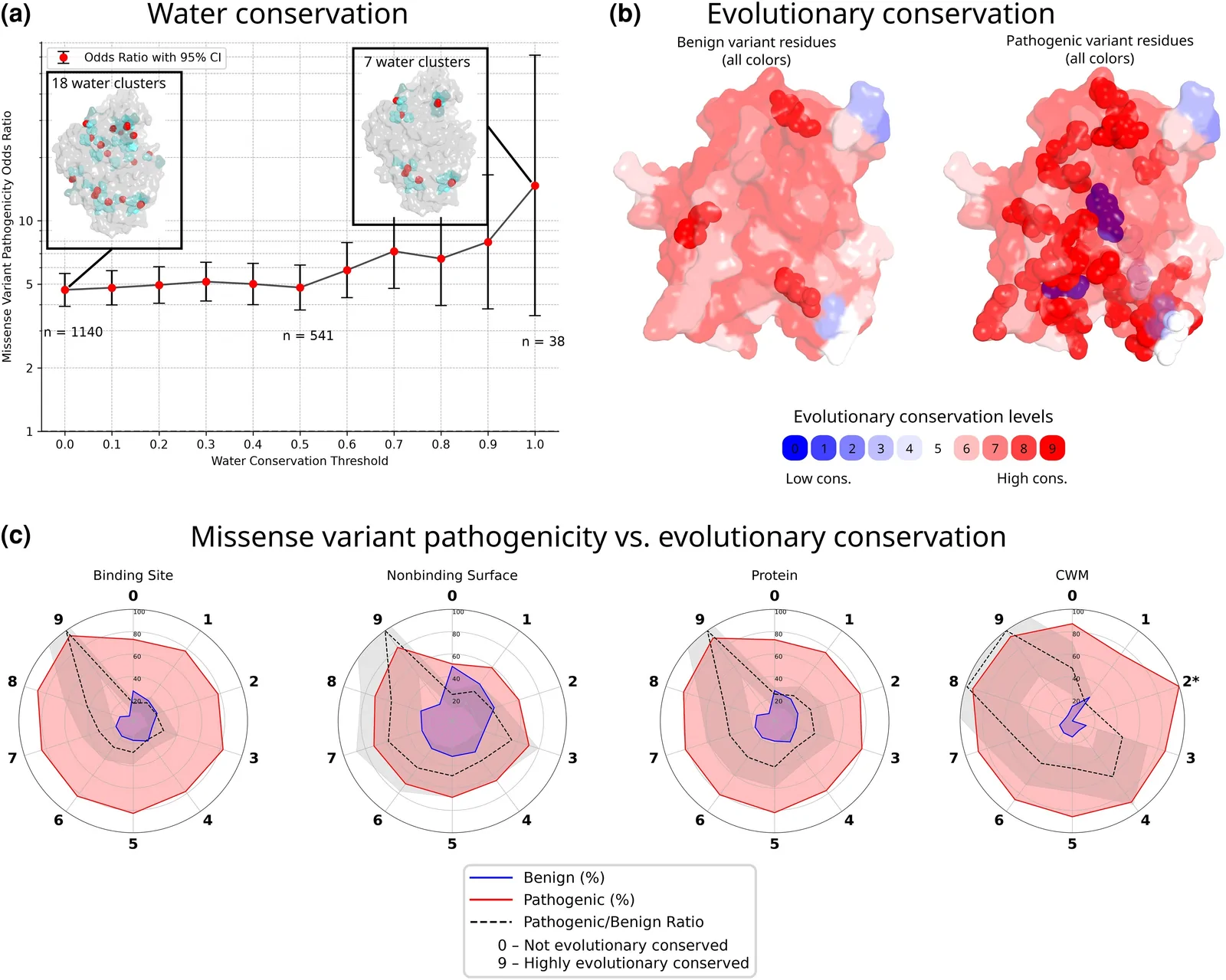
Influence of water and evolutionary conservation on missense variant pathogenicity. a) Odds ratios and 95% CIs for the association between pathogenic missense variants and CWM sites across water conservation thresholds. Each point includes CWM sites with conservation greater than or equal to the indicated threshold. Insets show predicted water clusters (red spheres) within CWM sites (cyan surface) in lysosomal acid glucosylceramidase (GCase; *GBA1*; PDB 1OGS) at selected thresholds. Number of pathogenic missense variants inside CWM sites, *n*. b) Evolutionary conservation mapped onto the surface of p53 (*TP53*; PDB 1TUP), ranging from 0, not conserved (blue), to 9, highly conserved (red). Residues harboring benign or pathogenic variants are shown as spheres and colored using the same scale. c) Radar plots showing the percentages of benign (blue) and pathogenic (red) missense variants and the normalized pathogenic-to-benign ratio (black dashed line; shaded 95% CI) across evolutionary conservation levels 0 to 9 in all binding-site classes combined, the nonbinding surface, protein-binding sites, and CWM sites. Asterisks indicate levels with missing data; for example, no benign missense variants were observed in CWM sites at conservation level 2.

### Effect of crystallographic resolution on CWM pathogenicity enrichment and water conservation

To assess resolution bias, we repeated the pathogenicity enrichment analysis for CWM and CWM-nonbinding sites using PDB structures with resolution either ≤ or ≥ each tested threshold. In the better-resolution subsets (≤ threshold), pathogenic variants remained significantly enriched across all thresholds. Odds ratios generally increased at more stringent cutoffs, particularly for CWM-nonbinding sites, although the trend was nonmonotonic and confidence intervals widened as sample sizes decreased. The complementary worse-resolution subsets (≥ threshold) showed no consistent trend and became increasingly imprecise at the poorest resolutions (SI, Figure S7a to d). Water conservation at CWM sites was negligibly correlated with the mean resolution of structures contributing waters to the cluster defining each site (Spearman ρ = −0.033; SI, Figure S7e).

### Comparison of experimentally resolved CWM-site waters in paired wild-type and variant PDB structures

To assess whether experimentally determined PDB structures are consistent with perturbation of predicted CWM sites by amino-acid substitutions, we analyzed 44 matched wild-type–variant structure pairs (SI, Table S1). In each pair, a CWM site was predicted in at least one structure, and the variant residue was located within that site. CWM sites were predicted in 42 wild-type and 22 variant structures. A matching experimentally resolved water was present at 38 of 42 predicted wild-type CWM sites (90.5%) and 17 of 22 predicted variant CWM sites (77.3%). Among the 38 pairs with a matching water in the wild-type structure, only 15 variant structures retained it. Two additional variant structures contained a matching water that was not resolved in the corresponding wild-type structure. The 3 benign variants showed unchanged experimental water status: 2 retained the water, whereas one lacked it in both structures.

### Conserved water molecules and pathogenic remodeling of lysosomal acid glucosylceramidase in Gaucher disease

Pathogenic variant residues were enriched at highly conserved CWM sites (water conservation ≥0.9) in lysosomal acid glucosylceramidase (GCase), including 3 variants at CWM sites overlapping other ligand-binding regions and twelve at CWM-nonbinding sites (Fig. 4a; SI, Figures S2 and S3, Dataset S1). The L444P variant (rs421016; p.Leu483Pro) mapped to a CWM-nonbinding site in domain II containing a conserved water that bridges 2 β-strands through up to 3 hydrogen bonds with Asp443 and Asp445 on one strand and Arg463 on the other (Fig. 4b). We therefore hypothesized that this CWM stabilizes the relative positioning of the opposing β-strands and that L444P disrupts this interaction.

**Figure 4:**
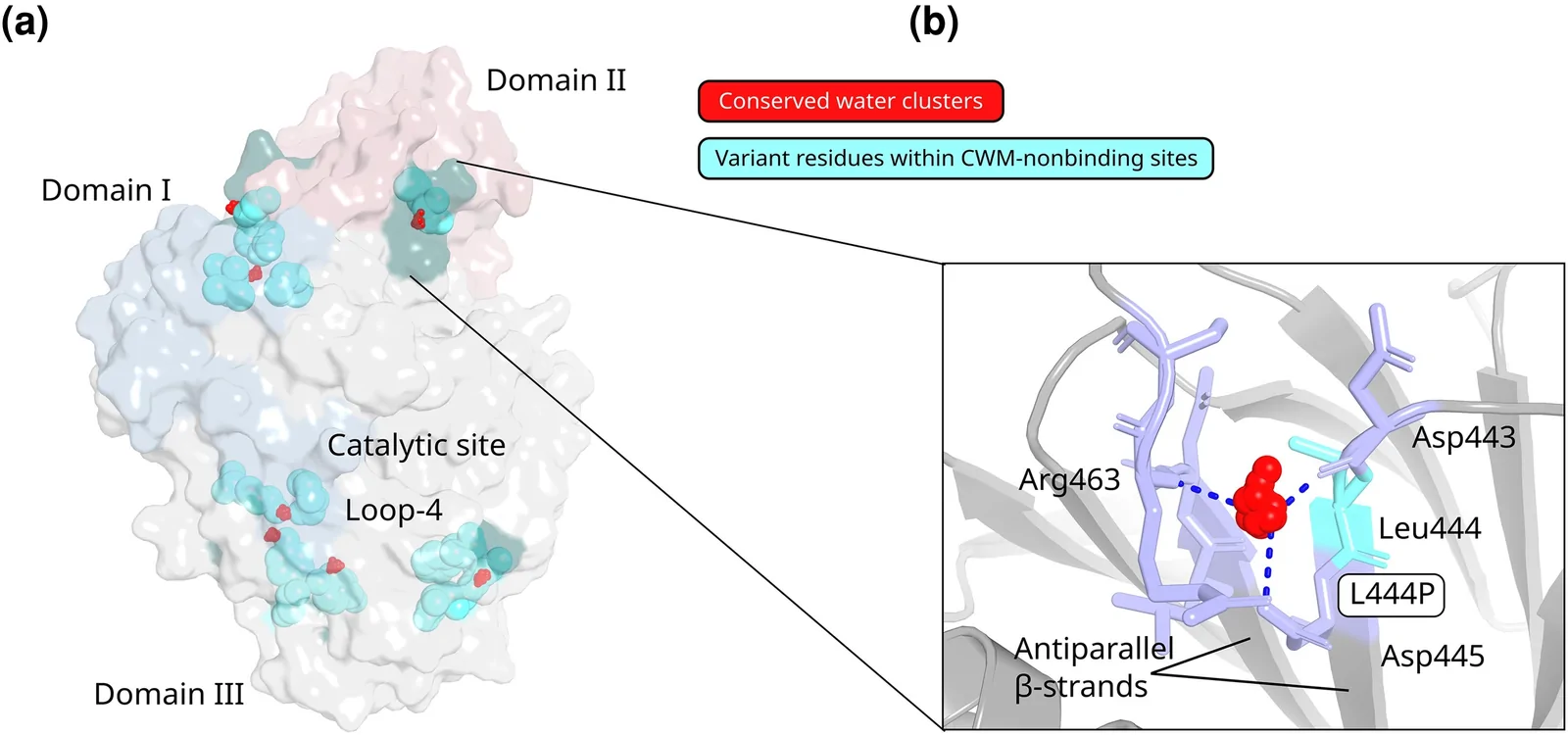
Pathogenic missense variant clusters in conserved water molecule (CWM) sites of lysosomal acid glucosylceramidase (GCase). a) Central view of GCase (PDB 1OGS) with CWM-nonbinding sites. Identified water clusters (water oxygen atoms, red spheres) are shown with variant residues (cyan spheres). b) Close-up of a CWM site (light purple sticks) with a water cluster (red spheres, water oxygen atoms only) near the Gaucher disease-causing L444P variant residue (rs421016; cyan sticks). Hydrogen bonds between clustered water molecules and protein residues are indicated by blue dashed lines.

To evaluate the structural consequences of CWM disruption, we performed three independent 1-µs molecular dynamics simulations for each of four systems: wild-type GCase, L444P, a CWM-knockout wild-type model, and rescued L444P. In the latter two systems, the identified CWM was selectively destabilized or stabilized, respectively. We quantified β-strand coupling as bridge occupancy: the percentage of frames containing at least one water molecule forming 2 or more protein hydrogen bonds, with at least one bond to each opposing β-strand.

Mean bridge occupancy was 68.67 ± 6.60% in wild-type GCase. It was lower in L444P (54.70 ± 0.95%; Fig. 5a) and the CWM knockout (37.24 ± 10.98%; Fig. 5b), although knockout occupancy varied substantially among replicates. CWM stabilization in rescued L444P modestly increased bridge occupancy to 58.48 ± 0.57% relative to L444P (Fig. 5c). Occupancies of bridging waters forming 2, 3, or 4 protein hydrogen bonds are reported in SI, Table S2.

**Figure 5:**
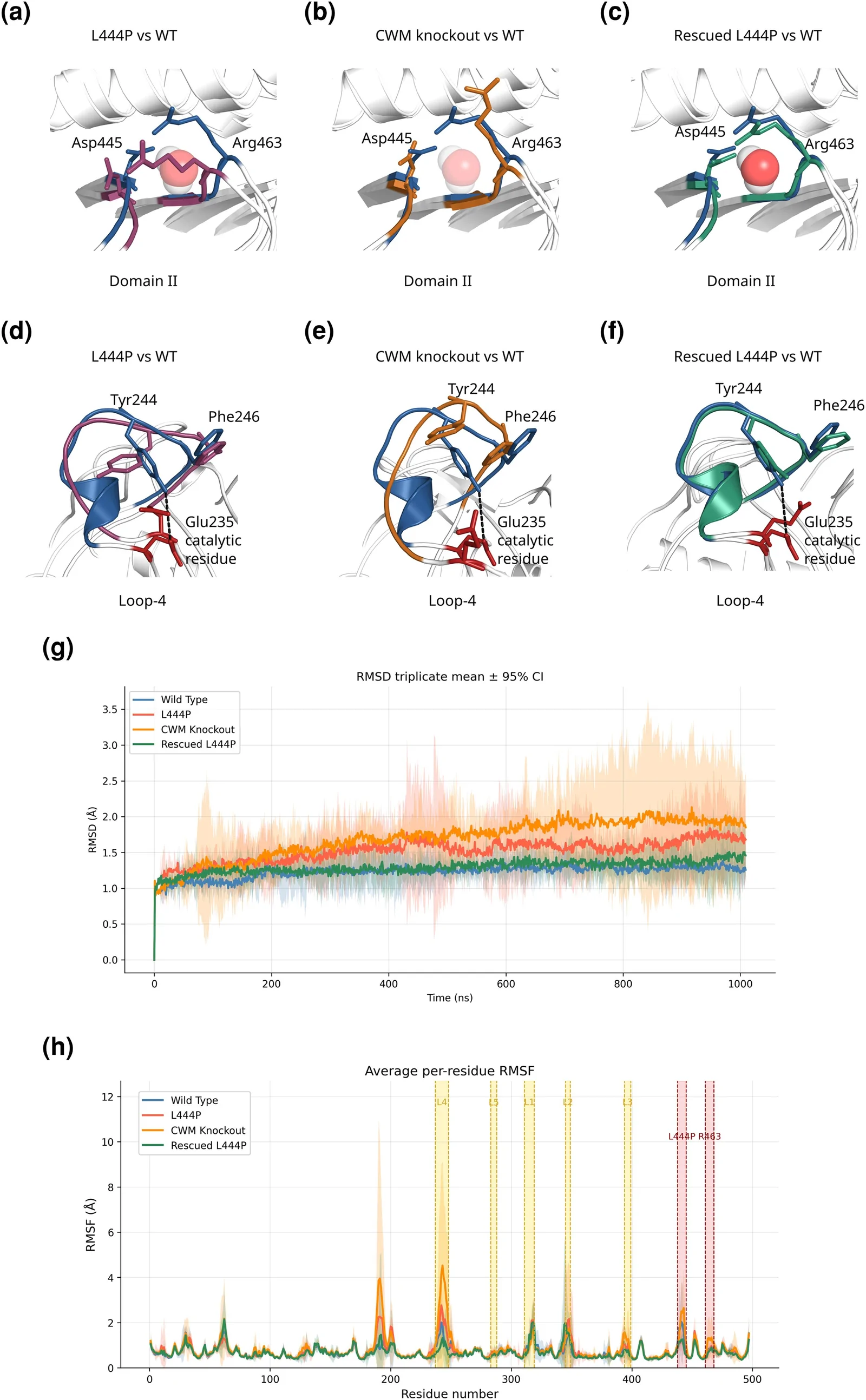
Dynamics of lysosomal acid glucosylceramidase (GCase) models relative to wild type. a to c) Representative structures of domain II antiparallel β-strands containing the identified conserved water molecule (CWM) for: (a) L444P (purple), (b) CWM knockout (orange), (c) rescued L444P (green), with CWM opacity reflecting water bridge occupancy (higher opacity, more wild-type–like). d to f) Representative structures of loop-4 for: (d) L444P, (e) CWM knockout, (f) rescued L444P. Wild-type structure is dark blue; catalytic Glu235 is red; Tyr244–Glu235 hydrogen bonds in wild-type are dashed lines. Representative structures in panels a–f are cluster medoids selected from the first of three independent replicate simulations. g) Mean Cα RMSD across three independent replicas for each GCase system. h) Mean per-residue Cα RMSF across three independent replicas. Shaded regions indicate 95% confidence intervals; vertical ribbons mark active-site-adjacent loops L1 to L5 (yellow) and residues surrounding the L444P/CWM site (pink).

In wild-type simulations, loop 4 adjacent to the active site adopted a dominant conformation stabilized by a Tyr244–Glu235 hydrogen bond. L444P and the CWM knockout sampled an additional conformation in which Tyr244 rotated away from catalytic Glu235 and the short α-helix spanning residues 237 to 241 was partially disrupted (Fig. 5d and e). Stabilizing the CWM in rescued L444P shifted local and global dynamics toward wild-type behavior, including partial recovery of the loop-4 conformation and Tyr244–Glu235 hydrogen bonding (Fig. 5c and f; SI, Figures S4 to S6).

Root mean square deviation (RMSD) profiles indicated greater overall structural stability in wild-type and rescued L444P, which generally showed lower RMSD values than L444P and the CWM knockout (Fig. 5g; SI, Figure S4). Root mean square fluctuation (RMSF) profiles further showed increased local flexibility in L444P and the CWM knockout, particularly in loops surrounding the active site, including loop 4, while rescued L444P partially reduced these fluctuations (residues 237 to 248; Fig. 5h; SI, Figure S6). As a complementary measure of local β-strand geometry, Asp445–Arg463 side-chain distance distributions varied among replicates and did not show a simple restoration toward the wild-type distribution in the rescued simulation (SI, Figure S5).

## Discussion

This study presents a proteome-wide structural analysis of pathogenic missense variants across protein binding sites, classified by ligand type and explicitly incorporating conserved water molecules (CWMs). Variant residues within binding sites of all ligand types combined were nearly three-fold more likely to be pathogenic than those on nonbinding protein surfaces (odds ratio [OR] 2.63; Fig. 1c), consistent with prior work (David et al. 2012; Das et al. 2014).

Pathogenic enrichment varied substantially across binding site classes (Fig. 2c). Metal ion and cofactor sites showed the strongest associations (ORs 8.52 and 6.55, respectively), reflecting stringent physicochemical constraints, whereas protein–protein interfaces exhibited more modest enrichment (OR 2.45), consistent with greater mutational tolerance due to larger interface size and partner co-evolution (Caffrey et al. 2004; Mintseris and Weng 2005). Glycan binding sites showed no significant enrichment (OR 1.09), likely due to smaller sample size or differences in site definition (Feinberg et al. 2013; Angata 2014).

A central finding is the strong and independent association between CWMs and missense variant pathogenicity. Both CWM sites that occur anywhere on a protein surface and CWM sites on otherwise ligand-free surfaces (CWM-nonbinding sites) were highly enriched for disease-causing variants (ORs 5.83 and 4.11, respectively; Fig. 2c), and their pathogenicity increased with water and evolutionary conservation (Fig. 3). These results confirm established roles of conserved water in protein stability and ligand recognition (Knight et al. 2009) and provide, to our knowledge, the first evidence for PDB-scale association between CWMs and human genetic disease.

To test whether the CWM association was robust to crystallographic resolution, we performed resolution-controlled analyses. Pathogenic enrichment remained significant across resolution thresholds, and CWM conservation was essentially independent of structure resolution (SI, Figure S7). Thus, the observed associations are unlikely to be driven by crystallographic resolution.

To determine whether the CWM association was supported by experimentally determined variant structures, we compared paired wild-type and variant PDB structures (SI, Table S1). A matching experimental water was resolved at 90.5% of predicted wild-type CWM sites but only 77.3% of predicted variant sites. Moreover, among 38 pairs containing the water in the wild-type structure, only 15 variant structures retained it. This pattern is consistent with substitutions weakening conserved water binding, although interpretation is constrained by the limited number of paired structures and by differences in the availability of structurally similar proteins for CWM prediction.

The L444P missense variant of lysosomal acid glucosylceramidase (GCase), a major determinant of Gaucher disease and a risk factor for Parkinson's disease, provides a mechanistic example of how CWM perturbation may contribute to pathogenic remodeling (Dvir et al. 2003). L444P maps to a highly conserved CWM-nonbinding site in domain II, where a single water bridges 2 antiparallel β-strands through hydrogen bonds involving residues Asp443–Leu444–Asp445 and Leu461–Asn462–Arg463 (Fig. 4).

Triplicate simulations showed reduced water-bridge occupancy and increased structural mobility in L444P and the CWM-knockout model, whereas CWM stabilization in rescued L444P shifted the dynamics partially toward wild-type behavior (Fig. 5; SI, Table S2). Our findings support a stabilizing role for the CWM but also indicate that its disruption may not be the sole cause of the L444P phenotype. In particular, L444P removes the Leu444 backbone amide hydrogen, disrupting its hydrogen bond with Asn442 and potentially remodeling the local Asn442–Leu444 segment, which may itself destabilize the adjacent CWM site.

The CWM destabilization propagated toward the active-site entrance, particularly loop 4 (Fig. 5e and h; residues 237 to 248), which contains Tyr244, an aromatic residue implicated in substrate recognition (Chi et al. 1999). Tyr244 lies near the catalytic residues Glu235 and Glu340, and its orientation influences active-site accessibility. Prior studies show that substitutions near the active site can alter the conformations of Tyr244 and Phe246 (Dvir et al. 2003), consistent with our observation that changes at the CWM site are accompanied by altered loop dynamics and Tyr244–Glu235 hydrogen bonding.

Residues 443 to 445 contribute to interactions with the facilitator protein saposin C (Romero et al. 2019), suggesting that local remodeling in this region due to weakened CWM binding could additionally affect GCase activation. Our findings are also consistent with observations in related β-glycosidases from hyperthermophilic organisms, where internal waters enhance protein stability (Chi et al. 1999). Our simulations identify CWM disruption as one component of a coupled local interaction network affected by L444P substitution, providing mechanistic context for therapeutic strategies aimed at restoring GCase stability and activity (Williams et al. 2024).

## Materials and methods

### Detection and classification of human protein binding sites by ligand type

Protein binding sites were identified using the GenProBiS web server (Konc and Janežič 2014; Konc et al. 2017) (http://genprobis.insilab.org). GenProBiS scans the entire PDB for local structural similarities and transfers co-crystallized ligands from matched template proteins to the query structure. It uses local structural alignment to identify equivalent surface regions in different proteins based on geometry and physicochemical properties that may contain bound ligands, including crystallographic waters. Ligands were classified into 7 types: cofactors, compounds, nucleic acids, proteins, glycans, ions, and conserved water molecules (CWMs). Nonspecific ligands and crystallographic artifacts were excluded using a curated list available at http://insilab.org/files/GenProBiS/non-specific.txt. Key steps were:

#### Step 1. Filtering for human structures

PDB protein chains were taxonomically filtered for *Homo sapiens*, yielding 120,628 chains for binding site and ligand detection.

#### Step 2. Template identification

Each human protein chain was used once as a query and compared against ∼320,000 PDB chains using the ProBiS algorithm (Konc and Janežič 2010), which detects local surface similarities across whole proteins. Similarity thresholds (Z-scores) were ligand-specific: >2.5 for compounds/cofactors, >3.0 for proteins/nucleic acids, and >2.0 for ions/water (Konc et al. 2017).

#### Step 3. Ligand transposition

Template ligands were mapped to the query via structural alignment if >3 residues (ions, water, small molecules) or >7 residues (proteins, nucleic acids) lay within 4 Å of any ligand atom. Ligands causing >10 atoms <1.0 Å steric clashes with the query were discarded.

#### Step 4. Ligand-binding and CWM site definition

Ligands were clustered by atomic proximity using the OPTICS algorithm (Ankerst et al. 1999). Each ligand cluster defined a distinct binding site, with qualifying water clusters designated as CWM sites. Ion and water clusters containing fewer than 10 members were excluded, and water clusters with conservation ≥0.6 were classified as CWMs (see below for the definition of water conservation). For each cluster, GenProBiS generated a hexagonal close-packed grid with 1.5 Å spacing. Grid points were retained if they were within 4 Å of a predicted ligand atom and within 8 Å of a query-protein atom. Residues with any atom within 3 Å of a retained grid point were classified as ligand-binding-site residues.

#### Step 5. Nonbinding protein surface definition

Nonbinding surface residues were defined as surface residues not assigned to any predicted binding site for compounds, cofactors, proteins, nucleic acids, glycans, or ions; residues assigned only to CWM-nonbinding sites were therefore included. Surface residues were defined as residues with at least one atom located ≤1.1 Å beneath the solvent-accessible surface, calculated using a 1.4 Å probe. For analyses involving water ligands, residues assigned to CWM-nonbinding sites were additionally excluded from the nonbinding protein surface, regardless of their water conservation score (see below). Thus, the resulting reference surface contained no residues assigned to any predicted CWM site.

### Water conservation at CWM sites

Water conservation quantifies the recurrence of crystallographic waters at structurally equivalent positions across locally aligned protein structures and was calculated as:


$$\mathrm{water}\;\mathrm{conservation}=\frac{\#\mathrm{structures}\;\mathrm{with}\;\mathrm{water}}{\#\mathrm{total}\;\mathrm{aligned}\;\mathrm{structures}}$$

Scores range from 0 (unconserved) to 1 (highly conserved) (Jukic et al. 2017). For each variant residue assigned to a CWM site, we used the highest water conservation score among the corresponding sites identified across structures containing that residue. Sites with scores ≥0.6 were classified as conserved protein-bound-water sites rather than random bulk solvent. Because water detection and modeling depend on crystallographic resolution, refinement, and local order, the score reflects conservation of observed water occupancy and does not require the water to be resolved in every individual structure.

### Mapping missense variants to binding sites

Missense variants from dbSNP (Phan et al. 2025) were mapped to protein structures using GenProBiS (Konc et al. 2017). A missense variant was classified as inside a binding site when the affected variant residue was assigned to a GenProBiS ligand-binding site (see Step 4), and as outside when it was at a nonbinding protein surface (see Step 5). ClinVar annotations were grouped as pathogenic (pathogenic or likely pathogenic) or benign (benign or likely benign); all other classifications were excluded (Landrum et al. 2025). Evolutionary conservation scores (0 to 9) for protein surface residues were computed from local structural alignments (Konc and Janežič 2012).

### Eliminating SNP redundancy

To avoid overrepresentation from PDB redundancy, missense variants were deduplicated by their reference SNP identifiers (rsIDs), reducing counts from 93,646 to 6,110. Missense variants were further collapsed by sequence identity clusters (>30% identity, >20 residues), retaining only those from the chain with the most missense variants in each cluster, yielding 4,580 unique missense variants. For each missense variant, we recorded: rsID, pathogenicity, binding site type(s), nonbinding surface status, buriedness (buried variants were excluded from analysis but still recorded), PDB/Chain ID, gene name, evolutionary conservation, maximum water conservation (only for missense variants within CWM sites), amino acid change, and UniProt ID.

### Statistical analysis

Fisher's exact test was used to compare the frequency of pathogenic missense variants in ligand-binding sites versus nonbinding surface regions. Odds ratios were computed for each ligand type and for all sites combined. For CWM and CWM-nonbinding sites, odds ratios for missense variant pathogenicity were computed across water conservation thresholds (0.0 to 1.0 in 0.1 increments) using a modified definition of the nonbinding surface (see Step 5). A similar framework was applied to evolutionary conservation: missense variants above a conservation threshold were treated as positives, those below as negatives.

### Controls for crystallographic-resolution bias at CWM sites

To test whether pathogenicity enrichment at CWM and CWM-nonbinding sites was influenced by crystallographic resolution, we repeated the odds-ratio analyses across thresholds from 1.5 to 3.5 Å. At each threshold, we analyzed 2 complementary datasets containing structures with resolution ≤ or ≥ the threshold; structures without a reported resolution were excluded.

To test whether CWM conservation depended on resolution for CWM sites, we first reduced sequence redundancy by retaining one representative structure from each 30% sequence-identity cluster, selected as the structure with the best available resolution (lowest Å value). For each GenProBiS CWM cluster in these representative structures, we identified the locally aligned PDB chains whose experimentally resolved waters contributed to the cluster and calculated their mean crystallographic resolution. Mean resolution was then correlated with the corresponding water conservation score using Spearman's rank correlation.

### Comparison of experimentally resolved CWM-site waters in paired wild-type and variant PDB structures

Starting from SI, Dataset S1, which lists all analyzed missense variants by rsID together with their GenProBiS ligand-binding-site annotations, we selected rsIDs mapped to predicted CWM sites. For each rsID, we searched the entire PDB and retained all chains containing that variant annotation. This enabled the identification of wild-type and variant structures for the same rsID. Each PDB chain was classified as wild type or variant by comparing the experimentally observed residue with the reference and substituted amino acids specified by the variant. Chains were then paired by rsID, and only rsIDs represented by both wild-type and variant structures were included. Because GenProBiS binding-site and ligand prediction was performed independently for each PDB chain, the variant position could be assigned to a CWM site in one structure but not another, even when both represented the same protein. For each predicted CWM site, we examined the corresponding GenProBiS ligand-annotation file, which lists the waters contributing to the CWM cluster from locally aligned structures. A matching experimental water was recorded when at least one water assigned to the cluster originated from the PDB chain being evaluated; its PDB residue identifier was retained.

### Molecular dynamics simulations of lysosomal acid glucosylceramidase variants

Molecular dynamics simulations were performed on human lysosomal acid glucosylceramidase (GCase) using the crystal structure PDB 1OGS (chain A). Four GCase systems were simulated: (i) wild-type, (ii) L444P, (iii) wild-type with the conserved water molecule disrupted (“CWM knockout”), and (iv) L444P with the CWM stabilized (“rescued L444P”). For each system, 3 independent 1-µs replicate simulations were performed using independently randomized initial velocities (Knapp et al. 2018).

The L444P substitution was introduced using PDBFixer in OpenMM (Eastman et al. 2024). All systems were solvated in explicit TIP3P-FB water with a 1.0 nm padding and neutralized at 0.15 M ionic strength using K^+^ and Cl^—^ ions. Simulations employed the AMBER14 force field and particle mesh Ewald electrostatics with a 1.0 nm nonbonded cutoff. All systems were energy-minimized (5,000 steps), heated to 310.15 K over 10 ns under NPT conditions at 1 bar using a Langevin integrator, and simulated for 1 μs. The first 100 ns were discarded as equilibration, and the remaining 900 ns were used for analysis.

In the CWM knockout simulation, electrostatic interactions between bulk water and 2 protein backbone amide hydrogens (Asp445 and Arg463) were selectively removed using a custom nonbonded force in OpenMM. Electrostatic terms between these protein atoms and all water atoms were set to zero, while van der Waals interactions and all other protein electrostatics were preserved, thereby destabilizing the conserved water site without otherwise perturbing the wild-type protein structure.

In the rescued L444P simulation, electrostatic interactions anchoring the conserved water molecule at its binding site were selectively strengthened. This was achieved by scaling the electrostatic terms between the Asp443 backbone carbonyl oxygen and bulk water hydrogens, and between the Arg463 amide hydrogen and bulk water oxygens, by a factor of 1.1. To permit exchange of the conserved water molecule with bulk solvent, as observed in the wild-type simulation, this scaling was applied intermittently in 100,000-step cycles (25,000 steps on, 75,000 steps off), using the L444P structure as the starting model.

Different atom pairs were used in knockout and rescue simulations because both had distinct aims. The knockout weakened the principal Asp445 and Arg463 backbone amide interactions to destabilize CWM occupancy. Strengthening these same interactions in preliminary rescue tests stabilized 2 separate waters, one bound to each β-strand, rather than a single bridge water. We therefore enhanced the Asp443 carbonyl oxygen–water hydrogen and Arg463 amide hydrogen–water oxygen interactions, favoring one water between the β-strands while allowing exchange with bulk solvent during unscaled intervals.

### Trajectory analyses

Trajectory analyses were performed using MDAnalysis (Naughton et al. 2022) for each replica separately and then averaged across replicas. Hydrogen bonds between water molecules and protein residues were identified using the geometric criteria implemented in the HydrogenBondAnalysis module in MDAnalysis and sampled every 100 trajectory frames over the final 900 ns of each simulation. For each sampled frame, protein hydrogen bonds were grouped by water oxygen atom. A water molecule was classified as bridging when it formed at least 2 protein hydrogen bonds, including at least one hydrogen bond with residues 443, 444, or 445 and at least one with residues 461, 462, or 463.

Overall trajectory bridge occupancy was defined as the percentage of sampled frames containing at least one bridging water. Multiplicity-specific trajectory occupancies were calculated as the percentages of sampled frames containing at least one bridging water forming exactly 2, 3, or 4 hydrogen bonds with the protein. Multiple bridging waters could occur in the same frame; consequently, a frame could contribute to more than one multiplicity category. Occupancies were calculated separately for each independent replicate and are reported as the mean ± sample standard deviation across 3 simulations.

Conformational clustering was performed on superposed trajectories of the first replica simulation aligned on protein Cα atoms using an automated k-means procedure with silhouette scoring to select the optimal number of clusters; representative structures were defined as cluster medoids.

## Supplementary Material

msag217_Supplementary_Data

## Data Availability

Data to generate missense variants annotated by their pathogenicity and involvement in CWM sites and specific ligand-binding sites for protein structures in the PDB and molecular dynamics trajectories of GCase variants are freely available at http://insilab.org/files/share/patho-snps-water-ligand-dataset.zip and at http://insilab.org/files/share/gcase-variants-md.zip.

## Materials and correspondence

Correspondence and material requests should be addressed to Janez Konc (janez.konc@ki.si).

## Code availability

Software developed for the statistical analysis and visualization of the missense variants mapped to ligand-binding sites and CWM sites is freely available at https://gitlab.com/janezkonc/patho-snp-water-ligand. Molecular dynamics scripts are available at https://gitlab.com/janezkonc/gaucher-md.

## References

[msag217-B1] Abramson J et al Accurate structure prediction of biomolecular interactions with AlphaFold 3. Nature. 2024:630:493–500. 10.1038/s41586-024-07487-w. PMC11168924 38718835

[msag217-B2] Angata T . Associations of genetic polymorphisms of Siglecs with human diseases. Glycobiology. 2014:24:785–793. 10.1093/glycob/cwu043. 24841380

[msag217-B3] Ankerst M, Breunig MM, Kriegel H-P, Sander J. OPTICS: ordering points to identify the clustering structure. SIGMOD Rec. 1999:28:49–60. 10.1145/304181.304187.

[msag217-B4] Auton A et al A global reference for human genetic variation. Nature. 2015:526:68–74. 10.1038/nature15393. PMC4750478 26432245

[msag217-B5] Backwell L, Marsh JA. Diverse molecular mechanisms underlying pathogenic protein mutations: beyond the loss-of-function paradigm. Annu. Rev. Genom. Hum. Genet. 2022:23:475–498. 10.1146/annurev-genom-111221-103208. 35395171

[msag217-B6] Ball P . Water is an active matrix of life for cell and molecular biology. Proc Natl Acad Sci U S A. 2017:114:13327–13335. 10.1073/pnas.1703781114. PMC5754758 28592654

[msag217-B7] Burke DF et al Towards a structurally resolved human protein interaction network. Nat Struct Mol Biol. 2023:30:216–225. 10.1038/s41594-022-00910-8. PMC9935395 36690744

[msag217-B8] Burley SK et al Updated resources for exploring experimentally-determined PDB structures and computed structure models at the RCSB protein data bank. Nucleic Acids Res. 2025:53:D564–D574. 10.1093/nar/gkae1091. PMC11701563 39607707

[msag217-B9] Caffrey DR, Somaroo S, Hughes JD, Mintseris J, Huang ES. Are protein–protein interfaces more conserved in sequence than the rest of the protein surface? Protein Sci. 2004:13:190–202. 10.1110/ps.03323604. PMC2286531 14691234

[msag217-B10] Campomizzi CS, Uttamrao PP, Stallone JJ, Rathinavelan T, Estrada DF. Asparagine-85 stabilizes a structural active site water network in CYP121A1 of Mycobacterium tuberculosis. Biochemistry. 2024:63:711–722. 10.1021/acs.biochem.3c00555. 38380587

[msag217-B11] Cheng J et al Accurate proteome-wide missense variant effect prediction with AlphaMissense. Science. 2023:381:eadg7492. 10.1126/science.adg7492. 37733863

[msag217-B12] Chi Y-I et al Crystal structure of the β-glycosidase from the hyperthermophile *Thermosphaera aggregans*: insights into its activity and thermostability. FEBS Lett. 1999:445:375–383. 10.1016/S0014-5793(99)00090-3. 10094493

[msag217-B13] Das J et al Elucidating common structural features of human pathogenic variations using large-scale atomic-resolution protein networks. Hum Mutat. 2014:35:585–593. 10.1002/humu.22534. PMC4876038 24599843

[msag217-B14] David A, Razali R, Wass MN, Sternberg MJ. Protein–protein interaction sites are hot spots for disease-associated nonsynonymous SNPs. Hum Mutat. 2012:33:359–363. 10.1002/humu.21656. 22072597

[msag217-B15] David A, Sternberg MJE. The contribution of missense mutations in core and rim residues of protein–protein interfaces to human disease. J Mol Biol. 2015:427:2886–2898. 10.1016/j.jmb.2015.07.004. PMC4548493 26173036

[msag217-B16] Dvir H et al X-ray structure of human acid-β-glucosidase, the defective enzyme in Gaucher disease. EMBO Rep. 2003:4:704–709. 10.1038/sj.embor.embor873. PMC1326319 12792654

[msag217-B17] Dyer SC et al Ensembl 2025. Nucleic Acids Res. 2025:53:D948–D957. 10.1093/nar/gkae1071. PMC11701638 39656687

[msag217-B18] Eastman P et al OpenMM 8: molecular dynamics simulation with machine learning potentials. J Phys Chem B. 2024:128:109–116. 10.1021/acs.jpcb.3c06662. PMC10846090 38154096

[msag217-B19] Feinberg H et al Common polymorphisms in human langerin change specificity for glycan ligands. J Biol Chem. 2013:288:36762–36771. 10.1074/jbc.M113.528000. PMC3873535 24217250

[msag217-B20] Gibbs RA et al The international HapMap project. Nature. 2003:426:789–796. 10.1038/nature02168. 14685227

[msag217-B21] Jubb HC et al Mutations at protein-protein interfaces: small changes over big surfaces have large impacts on human health. Prog Biophys Mol Biol. 2017:128:3–13. 10.1016/j.pbiomolbio.2016.10.002. 27913149

[msag217-B22] Jukic M, Konc J, Gobec S, Janezic D. Identification of conserved water sites in protein structures for drug design. J Chem Inf Model. 2017:57:3094–3103. 10.1021/acs.jcim.7b00443. 29155577

[msag217-B23] Jumper J et al Highly accurate protein structure prediction with AlphaFold. Nature. 2021:596:583–589. 10.1038/s41586-021-03819-2. PMC8371605 34265844

[msag217-B24] Kamburov A et al Comprehensive assessment of cancer missense mutation clustering in protein structures. Proc Natl Acad Sci U S A. 2015:112:E5486–E5495. 10.1073/pnas.1516373112. PMC4603469 26392535

[msag217-B25] Karczewski KJ et al The mutational constraint spectrum quantified from variation in 141,456 humans. Nature. 2020:581:434–443. 10.1038/s41586-020-2308-7. PMC7334197 32461654

[msag217-B26] Knapp B, Ospina L, Deane CM. Avoiding false positive conclusions in molecular simulation: the importance of replicas. J Chem Theory Comput. 2018:14:6127–6138. 10.1021/acs.jctc.8b00391. 30354113

[msag217-B27] Knight JDR, Hamelberg D, McCammon JA, Kothary R. The role of conserved water molecules in the catalytic domain of protein kinases. Proteins. 2009:76:527–535. 10.1002/prot.22451. PMC3287957 19425109

[msag217-B28] Konc J, Janežič D. Probis algorithm for detection of structurally similar protein binding sites by local structural alignment. Bioinformatics. 2010:26:1160–1168. 10.1093/bioinformatics/btq100. PMC2859123 20305268

[msag217-B29] Konc J, Janežič D. ProBiS-2012: web server and web services for detection of structurally similar binding sites in proteins. Nucleic Acids Res. 2012:40:W214–W221. 10.1093/nar/gks435. PMC3394329 22600737

[msag217-B30] Konc J, Janežič D. ProBiS-ligands: a web server for prediction of ligands by examination of protein binding sites. Nucleic Acids Res. 2014:42:W215–W220. 10.1093/nar/gku460. PMC4086080 24861616

[msag217-B31] Konc J, Skrlj B, Erzen N, Kunej T, Janezic D. GenProBiS: web server for mapping of sequence variants to protein binding sites. Nucleic Acids Res. 2017:45:W253–W259. 10.1093/nar/gkx420. PMC5570222 28498966

[msag217-B32] Kucukkal TG, Petukh M, Li L, Alexov E. Structural and physico-chemical effects of disease and non-disease nsSNPs on proteins. Curr Opin Struct Biol. 2015:32:18–24. 10.1016/j.sbi.2015.01.003. PMC4511717 25658850

[msag217-B33] Lander ES et al Initial sequencing and analysis of the human genome. Nature. 2001:409:860–921. 10.1038/35057062. 11237011

[msag217-B34] Landrum MJ et al ClinVar: updates to support classifications of both germline and somatic variants. Nucleic Acids Res. 2025:53:D1313–D1321. 10.1093/nar/gkae1090. PMC11701624 39578691

[msag217-B35] Livesey BJ, Marsh JA. The properties of human disease mutations at protein interfaces. PLoS Comput Biol. 2022:18:e1009858. 10.1371/journal.pcbi.1009858. PMC8849535 35120134

[msag217-B36] Meyer MJ et al Interactome INSIDER: a structural interactome browser for genomic studies. Nat Methods. 2018:15:107–114. 10.1038/nmeth.4540. PMC6026581 29355848

[msag217-B37] Mintseris J, Weng Z. Structure, function, and evolution of transient and obligate protein–protein interactions. Proc Natl Acad Sci U S A. 2005:102:10930–10935. 10.1073/pnas.0502667102. PMC1182425 16043700

[msag217-B38] Naughton FB et al MDAnalysis 2.0 and beyond: fast and interoperable, community driven simulation analysis. Biophys J. 2022:121:272a–273a. 10.1016/j.bpj.2021.11.1368.

[msag217-B39] Phan L et al The evolution of dbSNP: 25 years of impact in genomic research. Nucleic Acids Res. 2025:53:D925–D931. 10.1093/nar/gkae977. PMC11701571 39530225

[msag217-B40] Porta-Pardo E et al Comparison of algorithms for the detection of cancer drivers at subgene resolution. Nat Methods. 2017:14:782–788. 10.1038/nmeth.4364. PMC5935266 28714987

[msag217-B41] Rodríguez-Almazán C et al Structural basis of human triosephosphate isomerase deficiency: mutation E104d is related to alterations of a conserved water network at the dimer interface. J Biol Chem. 2008:283:23254–23263. 10.1074/jbc.M802145200. 18562316

[msag217-B42] Rolland T et al A proteome-scale map of the human interactome network. Cell. 2014:159:1212–1226. 10.1016/j.cell.2014.10.050. PMC4266588 25416956

[msag217-B43] Romero R et al Mechanism of glucocerebrosidase activation and dysfunction in Gaucher disease unraveled by molecular dynamics and deep learning. Proc Natl Acad Sci U S A. 2019:116:5086–5095. 10.1073/pnas.1818411116. PMC6421449 30808805

[msag217-B44] Ruderfer DM et al Patterns of genic intolerance of rare copy number variation in 59,898 human exomes. Nat Genet. 2016:48:1107–1111. 10.1038/ng.3638. PMC5042837 27533299

[msag217-B45] Sahni N et al Widespread macromolecular interaction perturbations in human genetic disorders. Cell. 2015:161:647–660. 10.1016/j.cell.2015.04.013. PMC4441215 25910212

[msag217-B46] Setny P . Conserved internal hydration motifs in protein kinases. Proteins. 2020:88:1578–1591. 10.1002/prot.25977. 32666522

[msag217-B47] Shaltiel S, Cox S, Taylor SS. Conserved water molecules contribute to the extensive network of interactions at the active site of protein kinase A. Proc Natl Acad Sci U S A. 1998:95:484–491. 10.1073/pnas.95.2.484. PMC18446 9435218

[msag217-B48] Sivley RM, Dou X, Meiler J, Bush WS, Capra JA. Comprehensive analysis of constraint on the spatial distribution of missense variants in human protein structures. Am J Hum Genet. 2018:102:415–426. 10.1016/j.ajhg.2018.01.017. PMC5985282 29455857

[msag217-B49] Spyrakis F et al The roles of water in the protein matrix: a largely untapped resource for drug discovery. J Med Chem. 2017:60:6781–6827. 10.1021/acs.jmedchem.7b00057. 28475332

[msag217-B50] Taliun D et al Sequencing of 53,831 diverse genomes from the NHLBI TOPMed program. Nature. 2021:590:290–299. 10.1038/s41586-021-03205-y. PMC7875770 33568819

[msag217-B51] Williams D et al High-throughput screening for small-molecule stabilizers of misfolded glucocerebrosidase in Gaucher disease and Parkinson's disease. Proc Natl Acad Sci U S A. 2024:121:e2406009121. 10.1073/pnas.2406009121. PMC11494340 39388267

