# Supplementary material for: Conserved water molecules shape the pathogenicity of missense variants in human proteins
Source: Mol Biol Evol. 2026 Nov 4;43(11):msag217. doi: 10.1093/molbev/msag217 (PMC13733661; doi:10.1093/molbev/msag217)
Supplement: Supplementary file 1 [file msag217_supplementary_data.zip › SI-final.pdf]

Supporting Information

Conserved water molecules shape the pathogenicity of missense variants in human proteins

Janez Konc,[*] Karmen Recer, Tanja Kunej, Dušanka Janežič

* Janez Konc
Email: janez.konc@ki.si

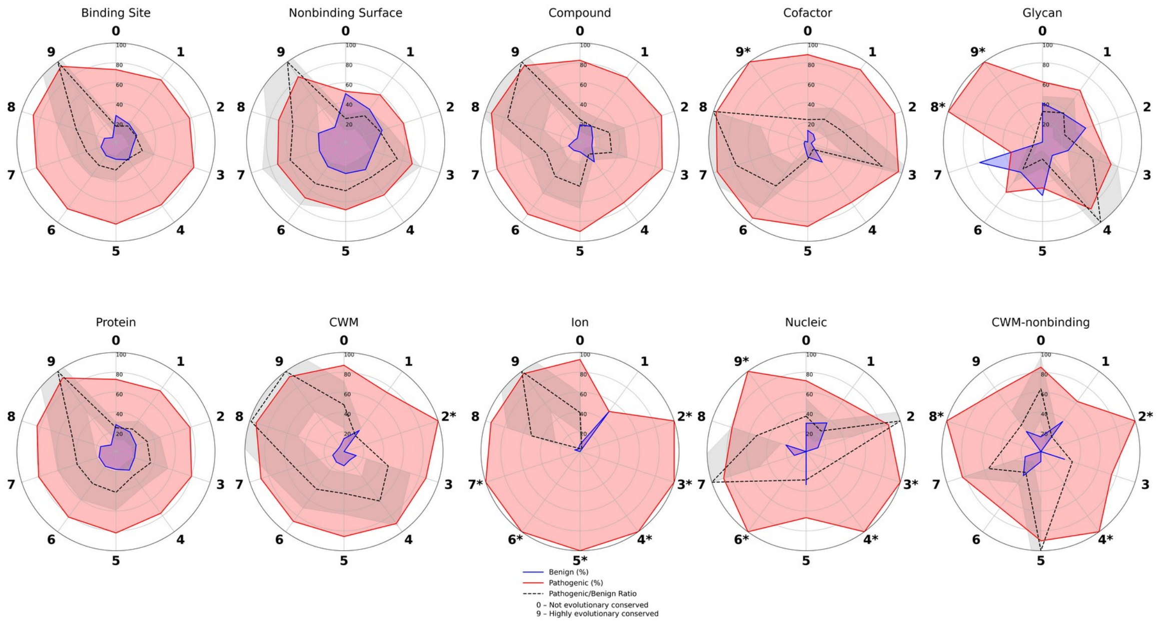

Fig. S1. Evolutionary conservation and missense variant pathogenicity across binding site types. Radar plots showing the percentage of benign (blue) and pathogenic (red) missense variants, and the normalized pathogenic-to-benign ratio (black dashed line, 95% CI shaded), across evolutionary conservation levels for all binding site types (labeled 0-9). Asterisks indicate conservation levels with missing data (e.g., no benign missense variants in CWM sites at level 2).

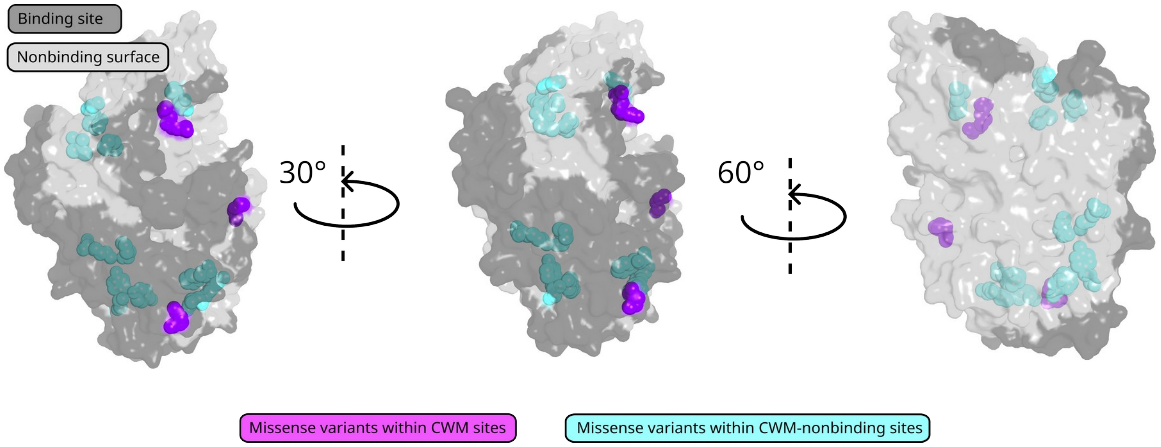

Fig. S2. Pathogenic variant residues within CWM sites and CWM-nonbinding sites (water conservation ≥ 0.9) mapped to the lysosomal acid glucosylceramidase (GCase) structure (*GBA1*; PDB 1OGS).

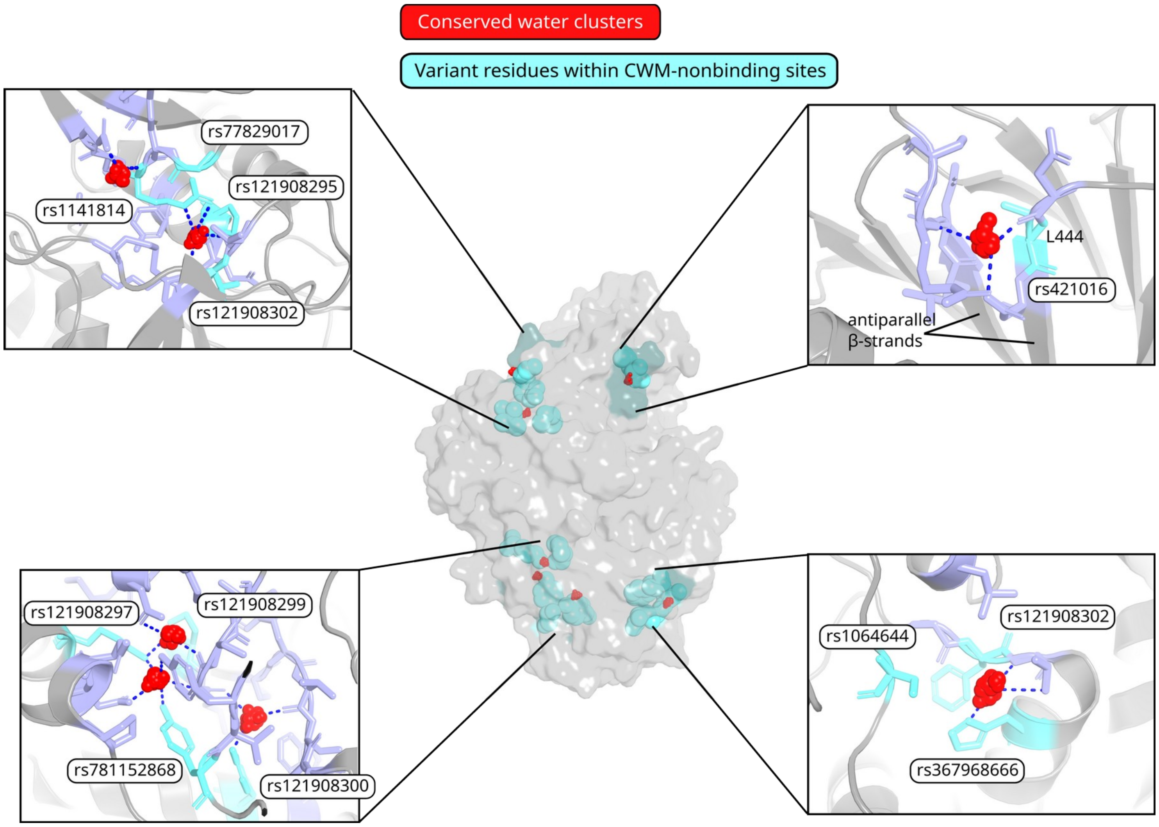

Fig. S3. Lysosomal acid glucosylceramidase (GCase) with close-ups of CWM-nonbinding sites. Conserved water clusters (red spheres, water oxygen atoms only) are shown with associated missense variant residues (cyan sticks) and other CWM-nonbinding site residues (light purple sticks). Hydrogen bonds between clustered water molecules and protein residues are indicated by blue dashed lines.

Replica 1

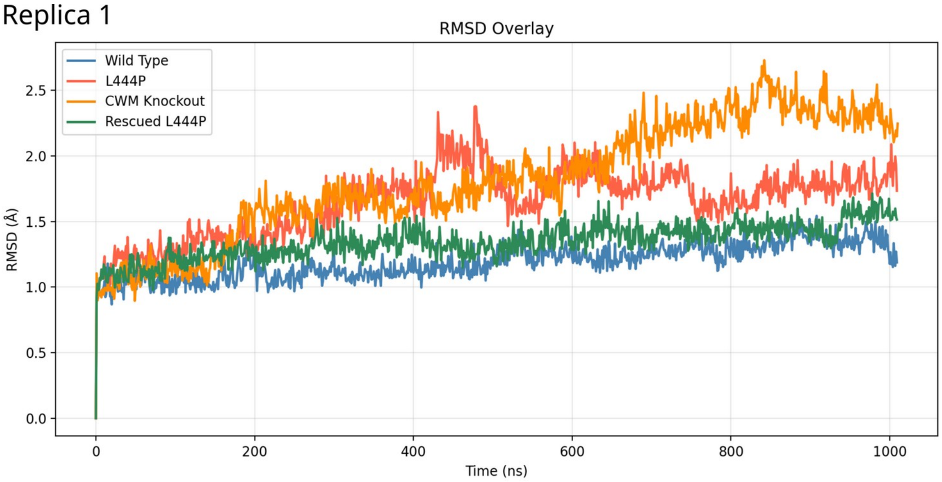

Replica 2

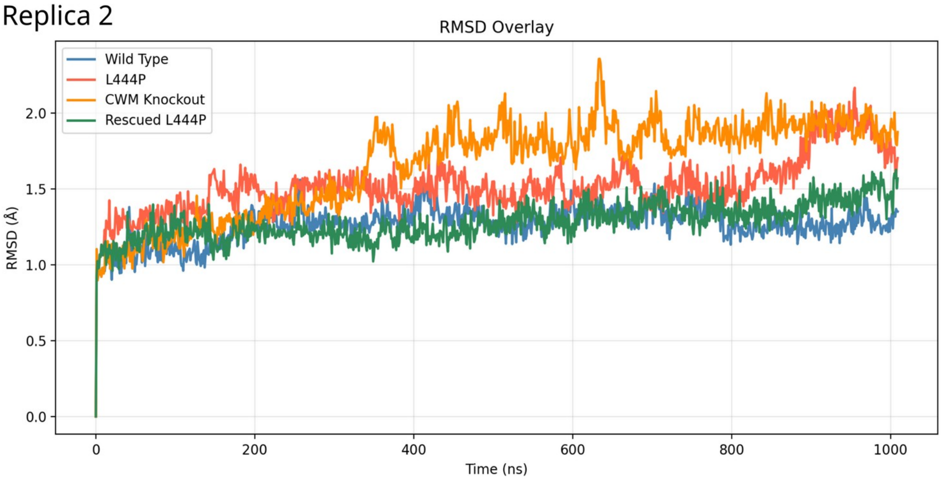

Replica 3

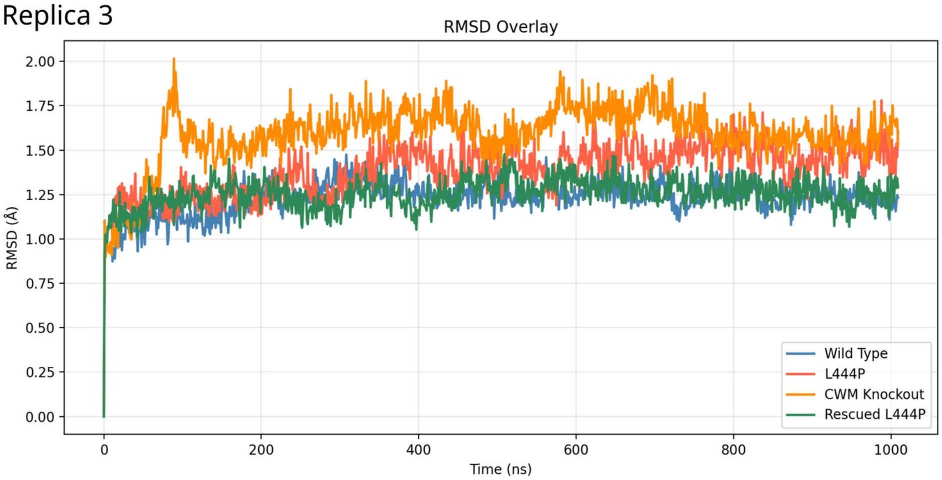

Fig. S4. Cα RMSD profiles of GCase variants for individual replicate simulations. Each panel shows one independent replica of wild type, L444P, CWM knockout, and rescued L444P.

Replica 1

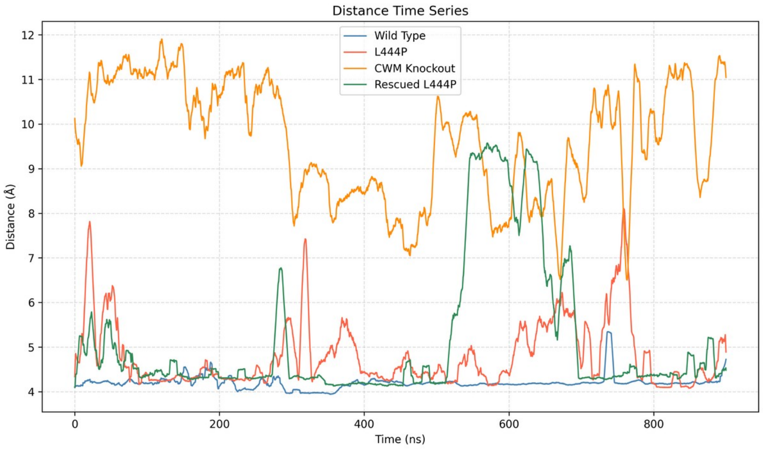

Replica 2

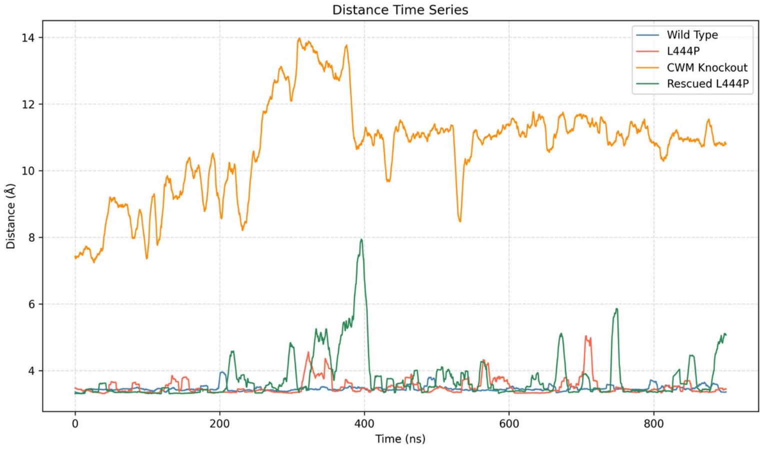

Replica 3

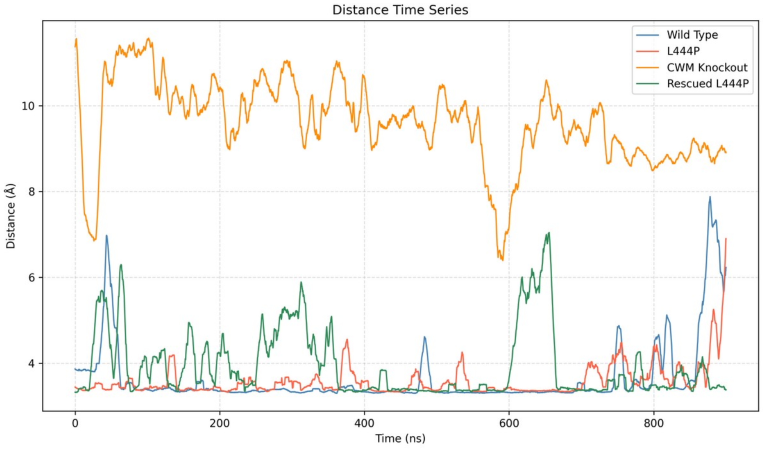

Fig. S5. Distance between Asp445 and Arg463 side chains in GCase variants across replicate simulations. This distance reports the local separation of the β-strand residues bridged by the conserved water molecule.

Replica 1

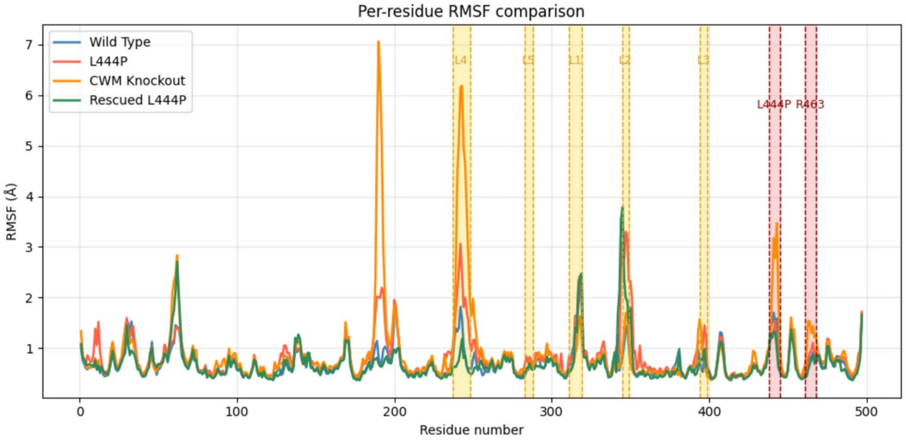

Replica 2

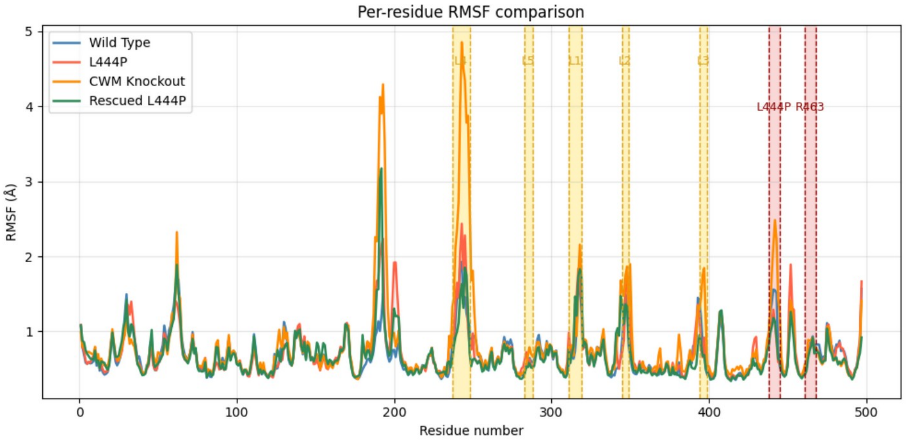

Replica 3

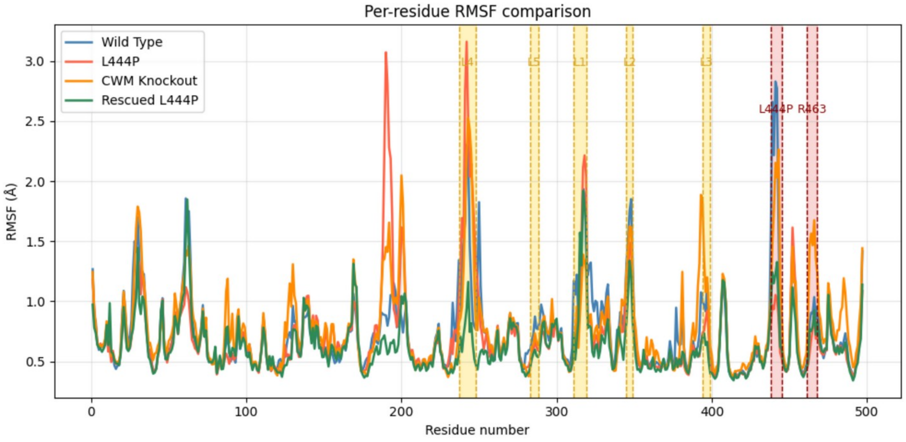

Fig. S6. Per-residue Cα RMSF profiles of GCase variants across replicate simulations. Highlighted regions indicate active-site-adjacent loops L1-L5 and residues surrounding the L444P/CWM site.

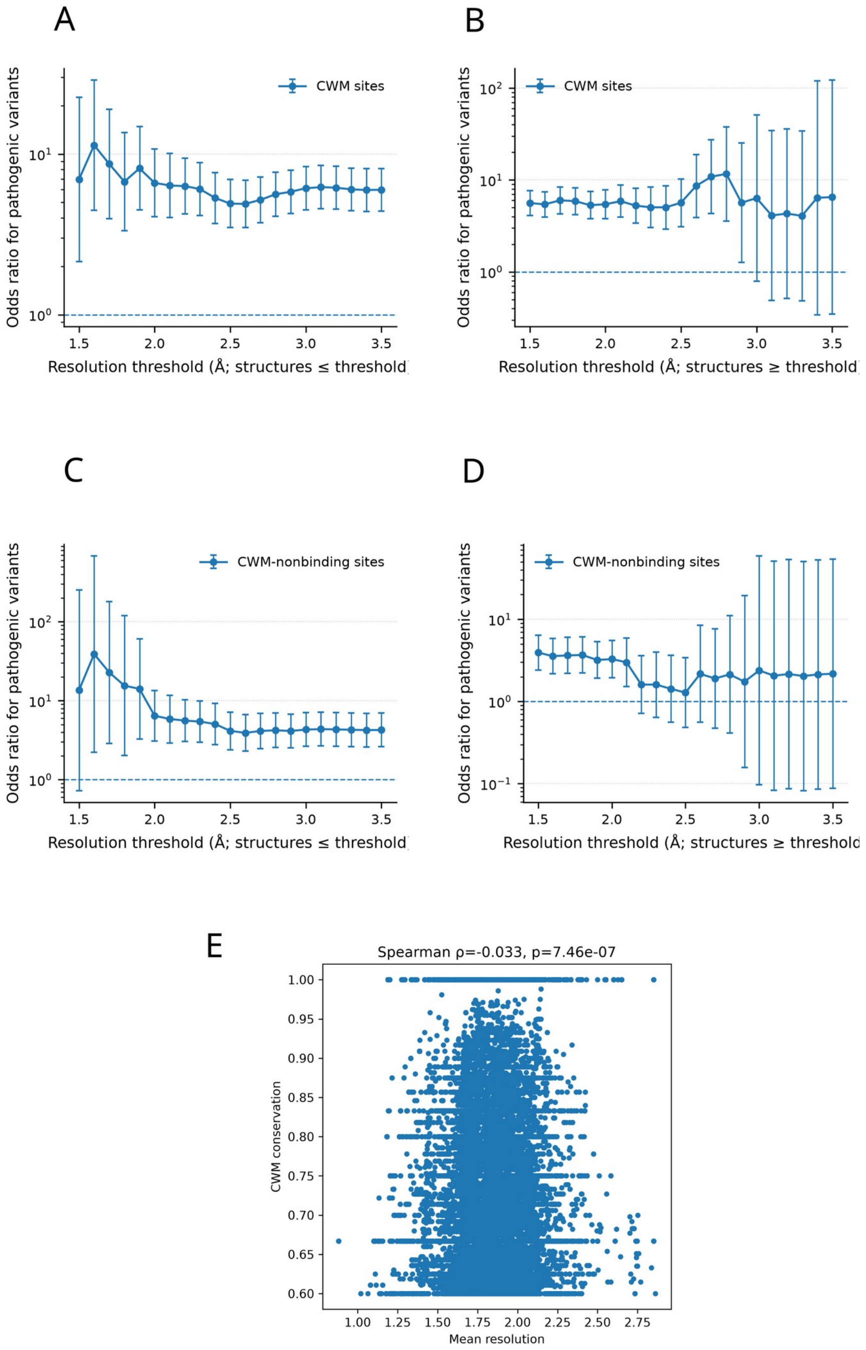

Fig. S7. Resolution-bias controls for CWM pathogenicity enrichment. Odds ratios for pathogenic variant enrichment at CWM sites after retaining structures with resolution (A) equal to or better than or (B) equal to or worse than each threshold, and at CWM-nonbinding sites using the corresponding (C) better-resolution and (D) worse-resolution filters. Lower Å values indicate higher crystallographic resolution. Error bars represent 95% confidence intervals; dashed lines indicate OR = 1. (E) Water conservation versus the mean resolution of locally aligned structures contributing waters to each GenProBiS CWM cluster.

Table S1. Experimentally resolved waters at predicted CWM sites in paired wild-type and variant PDB structures. Matched wild-type and variant structures are shown for missense variants whose substituted residue lies within a predicted CWM site in at least one structure.

			Wild-type structure			Variant structure		
rsID	Classification	Change	PDB_ chain	PDB water ID[1]	Water cons.[2]	PDB_ chain	PDB water ID[1]	Water cons.[2]
rs113994087	pathogenic	Arg1275Gln	4CD0_A	2041	0.682	4FNX_A		
rs119476044	pathogenic	Arg371His	3OUI_A	32	0.889	5LB6_A		
rs121434623	pathogenic	Lys61Arg	5HDE_A			5J8R_C	424	0.741
rs121908260	pathogenic	Arg46Gln	4AJX_D	2028	0.85	2MVD_B		
rs121909538	pathogenic	Arg55Lys	5EOP_A	253	0.761	4AHH_A	2013	0.727
rs121912451	pathogenic	Ser135Asn	1HL5_H		0.621	1OZU_A		
rs121912459	pathogenic	Asp97Asn	5J0F_A	362	0.651	3GTV_B		
rs121912666	pathogenic	Tyr220Cys	2AC0_D	320	0.9	5AOM_A	2176	0.85
rs121913343	pathogenic	Arg273Cys	2ADY_A	294	0.8	4IBU_A	425	0.6
rs121917852	pathogenic	Glu95Lys	3N1F_A	4	0.727	3K7H_B		
rs121917853	pathogenic	Glu131Lys	3N1O_A	198	0.636	3K7I_B		
rs121917854	pathogenic	Asp100Glu	3N1F_A	4	0.727	3K7J_B		
rs121918069	pathogenic	Leu78His	1BZE_A	205	0.836	3DJR_A		0.821
rs121918071	pathogenic	Ser97Tyr	4DEU_A	314	0.945	2TRY_A	135	0.768
rs121918072	pathogenic	Ile104Ser	1G1O_C	138	0.804	2NOY_A		
rs121918075	pathogenic	Tyr134Cys	4ABQ_A	2035	0.923	1IJN_A	906	0.855
rs121918079	pathogenic	Leu75Pro	3M1O_B	130	0.902	3DJZ_B	192	0.727
rs121918083	pathogenic	Cys30Arg	4IK7_B	330	0.942	2TRH_A		0.661
rs121918088	pathogenic	Tyr134His	4ABQ_A	2035	0.923	3DK2_A	136	0.873
rs121918101	pathogenic	His34Asp	5BOQ_B	115	0.737	1KMF_B		
rs121918102	pathogenic	Val92Leu	4GBC_C	101	0.615	2L1Z_A		1
rs121918456	pathogenic	Tyr279Cys	3ZM1_A	2052	0.776	4GWF_A		
rs121918468	pathogenic	Ala465Thr	3ZM1_A	2052	0.776	4OHD_A		
rs121918469	pathogenic	Gly468Ala	3ZM1_A	2271	0.621	4OHE_A		
rs121918552	pathogenic	Gly42Ser	5TY3_A	348	0.692	3NWV_A		
rs121964845	pathogenic	Glu142Asp	4UNK_B	2068	0.886	2VOM_A	2074	0.879
rs140547520	pathogenic	Glu117Gly	1FIK_A	206	0.75	4X1M_A		
rs1800458	benign	Gly26Ser	1TTB_B	409	0.691	1BZD_B	218	0.667
rs1800546	pathogenic	Ala150Pro	1QO5_C	2029	0.615	1XDL_A		
rs1800973	benign	Thr88Asn	1LZ4_A	172	0.923	1W08_A	2036	0.87
rs180177238	pathogenic	Ser187Phe	1H0C_A	2059	0.684	4I8A_A		
rs2304681	benign	Val49Ile	2HW1_A		0.667	2HLZ_A		
rs267606858	pathogenic	Thr83Met	3U2W_B			3RMV_A		0.615
rs28929474	pathogenic	Glu366Lys	1EZX_A	400	0.6	5IO1_A		
rs28933406	pathogenic	Gln61Lys	1XD2_A		0.69	2RGB_A		
rs28934574	pathogenic	Arg282Trp	4IBQ_A	505	0.675	2J21_A		
rs28934576	pathogenic	Arg273His	2ADY_A	294	0.8	4IBS_A	501	0.725
rs373849532	pathogenic	His112Asn	1KPE_A	127	0.897	5IPC_A	353	0.923
rs397515417	pathogenic	Thr311Met	3RQD_A	436	0.732	4QA3_A		0.696
rs41511344	pathogenic	Ser810Leu	2ABI_A		0.616	2OAX_A	7021	0.701
rs74315354	pathogenic	Glu163Lys	3CYF_A	201	0.889	2RK6_A	199	0.786

rs76992529	pathogenic	Val142Ile	3DGD_B	139	0.961	4HIS_B	318	0.873
rs80356668	pathogenic	Phe48Ser	1EV3_D	37	0.857	1HIQ_B		
rs80358193	pathogenic	Pro317Arg	4KBZ_A	605	0.889	5LAT_A	662	0.667

[1] PDB water ID denotes the PDB residue identifier of an experimentally resolved water matching the predicted CWM in the respective structure; an empty cell indicates that no matching water was identified.

[2] Water conservation is reported when a CWM site was predicted in that structure; an empty cell indicates that no CWM site was predicted.

Table S2. Trajectory occupancies of bridging water[1]-mediated hydrogen-bond states at the conserved water site near L444P in GCase.

System	Simulation	Any bridging water, ≥2 H-bonds (%)	Bridging water, exactly 2 H-bonds (%)	Bridging water, exactly 3 H-bonds (%)	Bridging water, exactly 4 H-bonds (%)
Wild type	1	69.56	54.67	17.44	0.00
	2	61.67	49.33	15.67	0.00
	3	74.78	66.44	13.11	0.00
	Mean ± SD	68.67 ± 6.60	56.81 ± 8.76	15.41 ± 2.18	0.00 ± 0.00
L444P	1	55.67	42.89	15.00	0.11
	2	53.78	30.00	25.00	0.00
	3	54.67	41.00	15.78	0.00
	Mean ± SD	54.70 ± 0.95	37.96 ± 6.96	18.59 ± 5.56	0.04 ± 0.06
CWM knockout	1	48.39	47.39	1.11	0.00
	2	36.89	35.56	1.33	0.00
	3	26.44	25.78	0.78	0.00
	Mean ± SD	37.24 ± 10.98	36.24 ± 10.82	1.07 ± 0.28	0.00 ± 0.00
Rescued L444P	1	58.33	45.22	14.44	0.00
	2	58.00	36.00	23.44	0.11
	3	59.11	42.00	19.22	0.00
	Mean ± SD	58.48 ± 0.57	41.07 ± 4.68	19.04 ± 4.50	0.04 ± 0.06

[1] A bridging water was defined as a water molecule forming at least two hydrogen bonds with the protein, including at least one hydrogen bond with residues 443–445 and at least one with residues 461–463. Overall trajectory bridge occupancy is the percentage of analyzed frames containing at least one bridging water. Multiplicity-specific occupancies are the percentages of analyzed frames containing at least one bridging water forming exactly two, three, or four protein hydrogen bonds. A single-H-bond category is not applicable because such a water cannot satisfy the bridge requirement. Because multiple bridging waters can coexist within the same frame, the multiplicity-specific categories are not mutually exclusive and their sum may exceed the overall bridge occupancy. Hydrogen bonds were sampled every 100 frames over the final 900 ns of each simulation. Values are shown for the three independent simulations and as mean ± sample SD.

Dataset S1 (separate file). Non-redundant missense variants mapped to CWM and ligand-binding sites. A total of 5,312 missense variants are annotated with pathogenicity, ligand-binding site classifications, evolutionary conservation, and structural context. Analysis was performed on the 4,580 missense variants located in binding sites or on the nonbinding protein surface; the remaining 732 buried missense variants (recognized by non-binding = 1 and non-binding surface = 0) were excluded. Data are organized in separate sheets according to water conservation thresholds (0.0-1.0).
